# A One-Pot Synthesis of Oxazepine-Quinazolinone bis-Heterocyclic Scaffolds via Isocyanide-Based Three-Component Reactions

**DOI:** 10.3389/fchem.2019.00623

**Published:** 2019-09-18

**Authors:** Shabnam Shaabani, Ahmad Shaabani, Monika Kucerakova, Michal Dusek

**Affiliations:** ^1^Faculty of Chemistry, Shahid Beheshti University, Tehran, Iran; ^2^Institute of Physics ASCR, Prague, Czechia

**Keywords:** oxazepine, quinazolinone, Ugi reaction, multicomponent reaction, isocyanide

## Abstract

A novel, efficient and environmentally friendly approach has been developed for the synthesis of biologically important *bis*-heterocyclic oxazepine-quinazolinone derivatives. The structurally interesting compounds of high purity were synthesized by a one-pot three-component reaction of 2-(2-formylphenoxy) acetic acid and 2-aminobenzamide as bifunctional reagents and an isocyanide without using any catalyst, with excellent overall yields.

## Introduction

To date, the development of new methods for the synthesis of heterocyclic compounds has been and remains a hot topic in organic chemistry, due to their importance in biologically active natural products and synthetic materials (Armstrong and Collins, 2010; Kaur et al., 2016). Remarkably, seven out of the top ten pharmaceutical products according to worldwide sales in 2009 contain a heterocyclic motif as their core structure (Chen et al., 2014). Seven-membered heterocyclic rings have been the object of deep investigation owing to their prevalence in molecules with biological activities (Goutham et al., 2015; Voigt et al., 2015; Xu, 2016).

**Figure 1:**
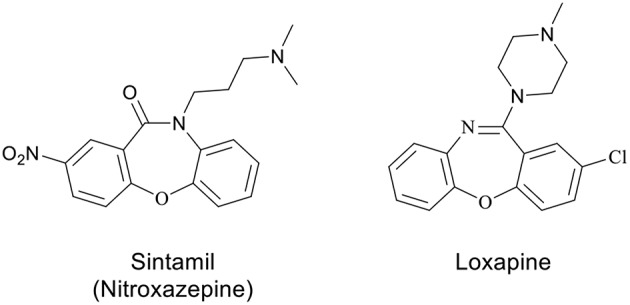
Examples of some biologically important oxazepines.

Oxazepines, a privileged scaffold in medicinal chemistry, are a well-known class of seven-membered heterocycles with two heteroatoms and have been receiving continuing attention due to the wide range of biological activities. Among these activities, it is worth mentioning anti-inflammatory (Chakrabarti and Hicks, 1987; Verma et al., 2008), antifungal (Serrano-Wu et al., 2002), antithrombotic (Mishra et al., 2010; Agirbas et al., 2011), anti-epileptic (Pekcec et al., 2009), anti-convulsant (Sharma et al., 2008), progesterone agonist (Dols et al., 2008), antagonist and analgesic (Hallinan et al., 1994), anti-histaminic (Sleevi et al., 1991), anti-psychotic (Liegeois et al., 1994; Liao et al., 1999), anxiolytics (Effland et al., 1982), anti-aggregating (Aono et al., 1991), and epidermal growth factor receptor (EGFR) tyrosine kinase inhibitory (Smith et al., 2006) activities. Compounds containing oxazepine motif, sintamil (Nagarajan et al., 1986) and loxapine (Liao et al., 1999) were reported, due to their antidepressant and potential clozapine-like properties, respectively (Figure 1) (Samet et al., 2005; Liu et al., 2011) Considering the structural characteristics of the benzoxazepine-3-ones, the existence of seven-membered heterocyclic ring system, fused aromatic group and the group –N–C(= O)–, similar to protein amide bond, it is reasonable to expect inherent physiological activities (Agirbas et al., 2011).

**Figure 2:**
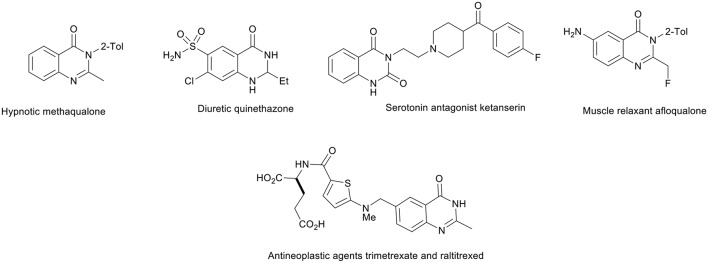
Structures of some quinazolinone derivative drugs.

Nitrogen heterocycles are the most important structural units in natural products and synthetic drugs. Thus, tremendous efforts have been made to develop new strategies and technologies for their synthesis (Tietze, 1996; Tietze and Modi, 2000; D'Souza and Mueller, 2007; Priebbenow et al., 2011; Rixson et al., 2012). Typically, quinazolinone derivatives widely occur in natural products (Yoshida et al., 1991; Wattanapiromsakul et al., 2003), and they show various biological and pharmacological activities, such as anti-inflammatory, antioxidant, antimicrobial, antipsychotic, and antihypertensive activity, strong analgesic activity, and many effects on the central nervous system (CNS) (Khalil et al., 1994; Bartoli et al., 1998; Liverton et al., 1998; Malecki et al., 2004; Arora et al., 2011; Chawla and Batra, 2013; Nepali et al., 2013). A quinazolinone motif is present in the structure of numerous drugs, e.g., the hypnotic methaqualone, the muscle relaxant afloqualone, the diuretic quinethazone, the antineoplastic agents trimetrexate and raltitrexed, and the serotonin antagonist ketanserin (Figure 2) (Kleemann et al., 1999; Abraham, 2003).

## Results and Discussion

**Scheme 1:**
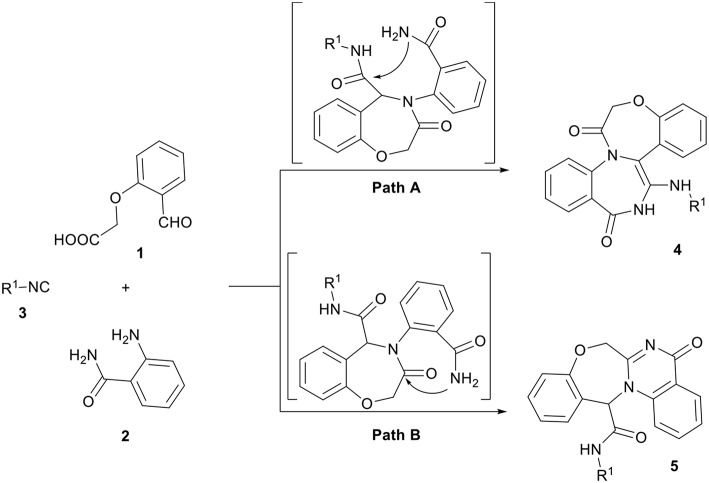
Intramolecular cyclization of Ugi product.

Combination of a molecule with several heterocyclic compounds with different pharmacological activities due to the synergism effect is a useful strategy to assign and discover new biological compounds. The Ugi four-component reaction (U-4CR) is one of the most commonly used multicomponent reactions (MCRs), in which a carboxylic acid, an amine, a carbonyl compound, and an isocyanide are reacting to result in peptide-like heterocyclic products (Hebach and Kazmaier, 2003; Dömling, 2006; Giovenzana et al., 2006; Ngouansavanh and Zhu, 2007; Hartweg and Becer, 2016; Yugandhar et al., 2016). Although a large diversity can be quickly achieved through the U-4CR, the scaffolds that are accessible through it are limited. The replacement of two participants in this reaction with a single bifunctional reagent is a fruitful strategy to broaden the scope of structures that are accessible by the U-4CR and toward various drug-like heterocycles (Hulme and Dietrich, 2009). 2-(2-formylphenoxy) acetic acid **1** has previously been employed to provide various derivatives of oxazepines (Zhang et al., 1999; Ilyin et al., 2006; Tsaloev et al., 2011; Hajishaabanha and Shaabani, 2014). As a part of our ongoing research program on the isocyanide-based MCRs (Shaabani et al., 2007, 2008a,b, 2009, 2011, 2014, 2016; Hajishaabanha and Shaabani, 2014), a novel strategy was designed to explore the Ugi one-pot three-component four-center reaction with two bifunctional starting materials, 2-(2-formylphenoxy)acetic acid **1** and 2-aminobenzamide **2** for the synthesis of *bis*-heterocyclic oxazepine-benzodiazepine **4** (Scheme 1, cyclization path A) or oxazepine-quinazolinone **5** (Scheme 1, cyclization path B) derivatives. The results show the reaction proceeded *via* the pathway B affording a new interesting class of oxazepine-quinazolinone **5** in high yields.

**Scheme 2:**
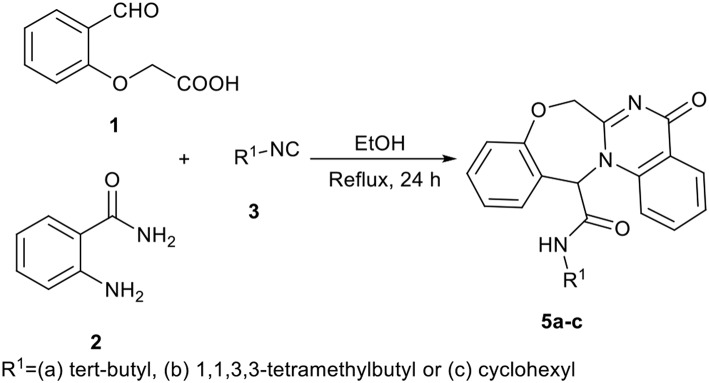
Synthesis of oxazepine-quinazolinone *bis*-heterocyclic scaffolds **5**.

In a pilot experiment, 2-(2-formylphenoxy)acetic acid **1**, 2-aminobenzamide **2**, and *tert*-butyl isocyanide **3a** were refluxed in ethanol. The progress of reaction was monitored by TLC. After 24 h, the reaction was completed and *N*-(*tert*-butyl)-5-oxo-5,7-dihydro-13*H*-benzo[6,7][1,4]oxazepino[4,3-a]quinazoline-13-carboxamide **5a** (Scheme 1, cyclization path B) was obtained in 94% yield (Scheme 2). It is worth mentioning that in the course of this reaction, one C-C bond, several C-N bonds, one amide group, a benzoxazepine ring and a quinazolinone ring are newly formed. These new structures broaden the scaffolds that are accessible through Ugi reaction and may represent interesting pharmacophores.

**Figure 3:**
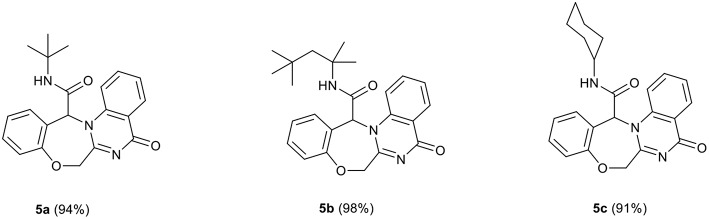
Structure and isolated yields of products **5**.

In view of the success of the above reaction, we explored its scope and limitations, by extending the procedure to various isocyanides **3a-c**. As indicated in Figure 3, the reactions proceed very efficiently in EtOH and led to the formation of novel oxazepine-quinazolinone *bis*-heterocyclic scaffolds **5a-c** in excellent yields. The reaction did not require any optimization.

**Figure 4:**
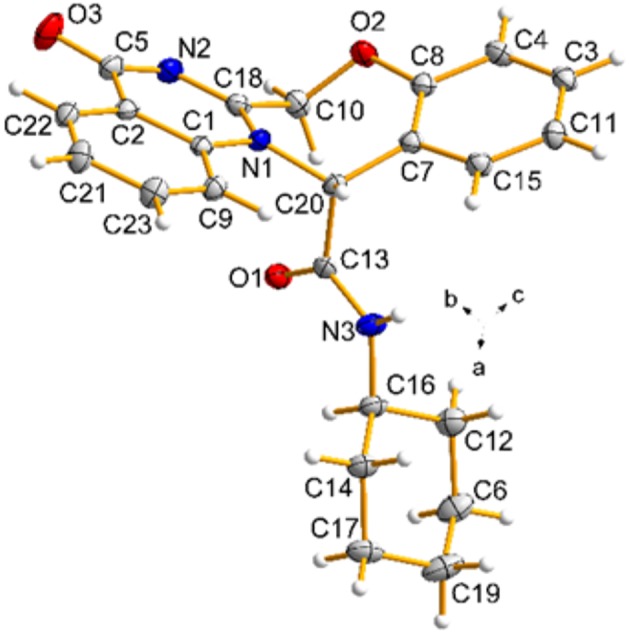
ORTEP diagram for **5c**.

The structures of products **5** were deduced from their IR, ^1^H NMR, ^13^C NMR, mass spectra and CHN analysis data. The ^1^H NMR spectrum of **5c** consisted of a multiplet for the methylene protons of the cyclohexyl ring (δ = 1.03-1.63 ppm, 10H), a broad singlet for the NH–C*H* cyclohexyl (δ = 3.58 ppm, 1H), two doublets for two non-equivalent methylene protons of the oxazepine ring (δ = 4.83 and 5.37 ppm, *J* = 14.5 Hz), a singlet for CH (δ = 6.65 ppm, 1H), a multiplet for aromatic protons and NH (7.03–8.11 ppm, 9H). Also, the ^1^H decoupled ^13^C NMR spectrum of **5c** is completely consistent with the suggested structure. The mass spectra of these compounds displayed molecular ion peaks at the appropriate *m/z* values. Finally, the structure of the product **5c** was confirmed unambiguously by single-crystal X-ray analysis (Figure 4) (Petríček et al., 2014).

**Scheme 3:**
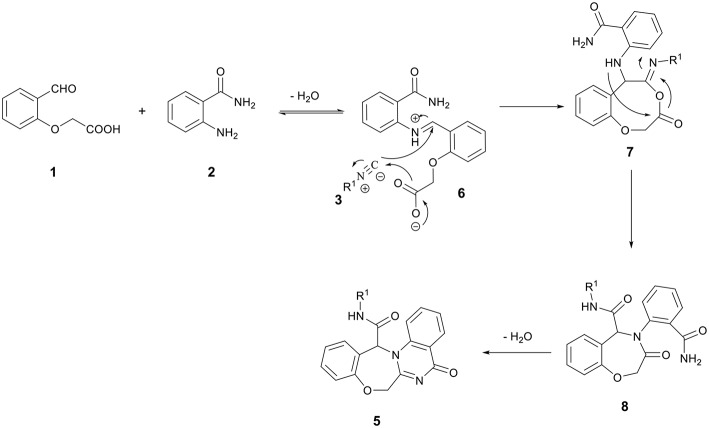
Proposed mechanism for the formation of products **5**.

A possible mechanism for the formation of products **5** is shown in Scheme 3. It is conceivable that the initial event in this reaction is the nucleophilic attack of amine **2** to formyl group to afford the iminium intermediate **6**. The addition of the carbenoid C-atom of the isocyanides **3** onto the iminium group followed by the addition of the carboxylate ion onto the C-atom of the nitrilium ion leads to the formation of the adduct **7**, which undergoes an intramolecular acylation known as Mumm rearrangement to give the Ugi adduct **8**. Finally, Ugi adduct **8** undergoes an amide-amide cyclocondensation through pathway A (instead of pathway B) to give the oxazepine-quinazolinone *bis*-heterocyclic products **5** (Scheme 3).

**Scheme 4:**
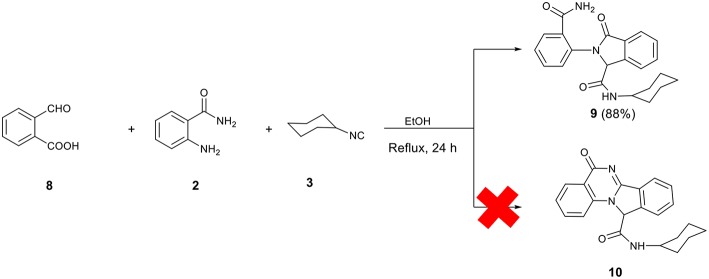
2-Formylbenzoic acid in Ugi 3-component reaction.

It is worth mentioning that to expand the structure diversity accessible through this type of Ugi 3-component reaction, the reaction between 2-formylbenzoic acid **8**, 2-aminobenzamide **2**, and cyclohexyl isocyanide under the previously mentioned conditions was also investigated. However, Ugi adduct **9** does not undergo an intramolecular cyclization to give the expected quinazolinone-isoindoline *bis*-heterocyclic product **10**. The analytical data obtained on the final material support the preparation and isolation of **9** as the product (Scheme 4).

**Figure 5:**
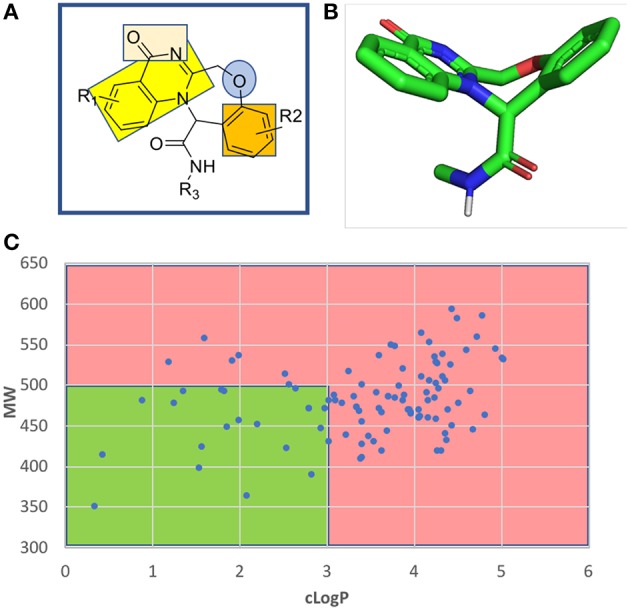
Chemoinformatic analysis of the unprecedented benzoxazepine-quinazolinone scaffold. **(A)** 2D structure and pharmacophores. **(B)** 3D energy minimized butterfly structure of the scaffold induced by the central seven-membered oxazepine ring. **(C)** Molecular weight over lipophilicity of a randomly generated a 100 compound library highlighting the drug-like preferred and non-preferred area in green and red, respectively.

The fused benzodiazepine-quinazolinone is a unique tetracyclic scaffold in several respects. A SciFinder and ChEMBL database search revealed no other example (Figure S1). Known substructures are benzoxazepines and quinazolinones. The parent scaffold benzoxazepine-quinazolinone is non planar through the introduction of the seven-membered aliphatic oxazepine ring in the center of the tetracycle, comprising a butterfly shape and showing an interesting combination of pharmacophores (Figure 5). The quinazolinone bicycle and the phenyl group are planar and can potentially undergo pi stacking interactions with the receptor amino acids. The quinazolinone also comprise a rare vicinal hydrogen bond acceptor hydrogen bond acceptor moiety. The other nitrogen atom is fully encapsulated in the ring systems and involved in the aromatic bicycle and cannot undergo hydrogen bonding interactions. The ether oxygen of the seven-membered oxazepane ring can act as another hydrogen bond acceptor.

A randomly generated library of benzoxazepine-quinazolinones reveals a good fraction of compounds with an attractive MW and cLogP thus rendering the scaffold interesting for receptor ligand interactions (Figure 5 and Supplementary Information).

## Conclusion

In conclusion, we have successfully developed a one-pot three-component four-center reaction strategy leading to novel *bis*-heterocyclic oxazepine-quinazolinones which are two important pharmacological and biological scaffolds, starting from simple and readily available inputs. To the best of our knowledge, it is the first report of using two bifunctional starting materials in Ugi reaction to obtain fused oxazepine-quinazolinone heterocycles. Moreover, it is a new isocyanide based bicyclization reaction (Gao et al., 2015, 2016; Hao et al., 2016; Tang et al., 2016). The reaction is high-yielding and product isolation is very straightforward. Moreover, it is noteworthy that this operationally friendly and scalable manner allows C–C bond, C-O and C–N bond formation with excellent scope. The potential uses of this route in synthetic and medicinal chemistry may be significant, since the products share structural and functional group properties of the biologically active molecules. Structural diversity and biological activity of the synthesized compounds will be tested and results of these tests will be reported in due course.

## Data Availability

All datasets generated for this study are included in the manuscript and/or the Supplementary Files.

## Author Contributions

SS did the design, synthesis, and wrote the manuscript. AS directed the project and co-wrote the manuscript. MK and MD did the crystallographic part.

### Conflict of Interest Statement

The authors declare that the research was conducted in the absence of any commercial or financial relationships that could be construed as a potential conflict of interest.
