# Supplementary material for: A One-Pot Synthesis of Oxazepine-Quinazolinone bis-Heterocyclic Scaffolds via Isocyanide-Based Three-Component Reactions
Source: Front Chem. 2019 Sep 18;7:623. doi: 10.3389/fchem.2019.00623 (PMC6759581; doi:10.3389/fchem.2019.00623)
Supplement: Supplementary file 1 [file Data_Sheet_1.doc]

*Supporting Information for*

A One-Pot Synthesis of Oxazepine-Quinazolinone bis-Heterocyclic Scaffolds via Isocyanide-Based Three-Component Reactions

Shabnam Shaabani,^1^ Ahmad Shaabani,^*1^ Monika Kucerakova^2^ and Michal Dusek^2^

^1^Department of Chemistry, Shahid Beheshti University, G. C., P. O. Box 19396-4716, Tehran*, Iran;* Fax: +982122431671; E-mail: [a-shaabani@sbu.ac.ir](mailto:a-shaabani@sbu.ac.ir).

^2^Institute of Physics ASCR, Na Slovance 2, 182 21 Prague, Czech Republic.

**Table of Contents**

| **List of contents** | **Page** |
| --- | --- |
| Title, authors’ name, address and tables | S1-S2 |
| Experimental section | S3-S5 |
| IR of **5a** | S6 |
| ^1^H NMR of **5a** | S7 |
| ^13^C NMR of **5a** | S8 |
| Mass of **5a** | S9 |
| IR of **5b** | S10 |
| ^1^H NMR of **5b** | S11 |
| ^13^C NMR of **5b** | S12 |
| Mass of **5b** | S13 |
| IR of **5c** | S14 |
| ^1^H NMR of **5c** | S15 |
| ^13^C NMR of **5c** | S16 |
| Mass of **5c** | S17 |
| IR of **9** | S18 |
| ^1^H NMR of **9** | S19 |
| ^13^C NMR of **9** | S20 |
| Mass of **9**  Chemoinformatic analysis  Virtual library synthesis | S21  S22  S23 |

EXPERIMENTAL PROCEDURES

General Information

Melting points were measured on an Electrothermal 9200 apparatus. IR spectra were recorded on a Shimadzu IR-470 spectrometer. ^1^H Spectra were recorded on a Bruker DRX-300 Avance spectrometer 300.13 MHz; chemical shifts (δ scale) are reported in parts per million (ppm). ^1^H NMR Spectra are reported in order: number of protons, multiplicity and approximate coupling constant (*J* value) in hertz (Hz); signals were characterized as s (singlet), d (doublet), t (triplet), m (multiplet), q (quartet) ,br s (broad signal) and Ar (aryl). The ^13^C NMR spectra were recorded at 75.47 MHz; chemical shifts (δ scale) are reported in parts per million (ppm). The mass spectra were recorded on an ionization potential of 70 eV. The elemental analyses were performed with an ElementarAnalysensysteme GmbH VarioEL. All the products are new compounds, which were characterized by IR, mass, ^1^H NMR and ^13^C NMR spectra.

General procedure

A mixture of 2-(2-formylphenoxy)acetic acid (0.18 g, 1 mmol), 2-aminobenzamide (0.13 g, 1 mmol) and an isocyanide (1 mmol) was refluxed in EtOH (5 ml) for 24 h. After completion of the reaction, as indicated by TLC (ethyl acetate/*n*-hexane, 3/1), the product was filtered off and washed with EtOH to give the pure product.

**Characterization Data**

***N*-(*tert*-butyl)-5-oxo-5,7-dihydro-13*H*-benzo[6,7][1,4]oxazepino[4,3-a]quinazoline-13-carboxamide 5a.** White powders: 341 mg, 94% yield; mp>270 °C. IR (KBr) cm^-1^: 3413, 3305, 2970, 1682, 1644, 1603, 1538. ^1^H NMR (300.13 MHz, DMSO-*d_6_*) δ: 1.20 (9H, s, C(CH3)3), 4.83 (1H, d, ^2^*J*_AB_ =14.2 Hz, O-CH_2_), 5.45 (1H, d, ^2^*J*_AB_ =14.2 Hz, O-CH_2_), 6.61 (1H, s, CH), 7.03-8.09 (9H, m, H-Ar and NH). ^13^C NMR (75.47 MHz, DMSO-*d_6_*) δ: 25.5, 52.0, 62.2, 72.8, 115.8, 120.2, 120.3, 122.5, 123.3, 126.7, 128.1, 131.2, 132.7, 134.8, 141.5, 156.5, 160.9, 167.0, 168.1. MS *m/z*: 364 (M^+^+1, 5), 263 (100), 247 (99), 195 (30), 167 (28), 77 (25), 41 (27). Anal. Calcd for C_21_H_21_N_3_O_3_: C, 69.41; H, 5.82; N, 11.56; found C, 69.37; H, 5.86; N, 11.44.

**5-Oxo-*N*-(2,4,4-trimethylpentan-2-yl)-5,7-dihydro-13*H*-benzo[6,7][1,4]oxazepino[4,3-*a*]quinazoline-13-carboxamide 5b.** White powders: 410 mg, 98% yield; mp 254 °C. IR (KBr) cm^-1^: 3427, 3278, 2954, 1688, 1637, 1603, 1537. ^1^H NMR (300.13 MHz, DMSO-*d_6_*) δ: 0.73 (9H, s, 3CH_3_), 1.26 (3H, s, CH_3_), 1.29 (3H, s, CH_3)_, 1.56 (2H, s, CH_2_), 4.86 (1H, d, ^2^*J*_AB_ =14.4 Hz, O-CH_2_), 5.50 (1H, d, ^2^*J*_AB_ =14.4 Hz, O-CH_2_), 6.64 (1H, s, CH), 7.06-8.13 (9H, m, H-Ar and NH). ^13^C NMR (75.47 MHz, DMSO-*d_6_*) δ: 28.3, 29.2, 31.4, 31.5, 51.6, 55.7, 55.8, 62.4, 73.1, 115.9, 120.2, 120.3, 122.4, 123.5, 126.8, 128.2, 131.3, 132.7, 134.8, 141.5, 156.8, 161.2, 166.4, 168.0. MS *m/z*: 420 (M^+^+1, 3), 264 (100), 247 (99), 195 (30), 167 (25), 77 (23), 41 (30). Anal. Calcd for C_25_H_29_N_3_O_3_: C, 71.57; H, 6.97; N, 10.02; found C, 71.52; H, 6.92; N, 10.05.

***N*-cyclohexyl-5-oxo-5,7-dihydro-13*H*-benzo[6,7][1,4]oxazepino[4,3-a]quinazoline-13-carboxamide 5c.** White powders: 353 mg, 91% yield; mp>270 °C. IR (KBr) cm^-1^: 3421, 3296, 2935, 2856, 1680, 1643, 1603, 1537. ^1^H NMR (300.13 MHz, DMSO-*d_6_*) δ: 1.03-1.63 (10H, m, 5CH_2_ of cyclohexyl), 3.58 (1H, br s, NH-C*H* of cyclohexyl), 4.83 (1H, d, ^2^*J*_AB_ =14.5 Hz, O-CH_2_), 5.37 (1H, d, ^2^*J*_AB_ =14.5 Hz, O-CH_2_), 6.65 (1H, s, CH), 7.03-8.11 (9H, m, H-Ar and NH). ^13^C NMR (75.47 MHz, DMSO-*d_6_*) δ: 25.1, 25.5, 32.1, 49.4, 61.9, 71.3, 115.9, 119.9, 120.4, 122.5, 123.2, 126.7, 128.1, 131.3, 133.3, 134.7, 141.5, 156.3, 160.3, 167.0. MS *m/z*: 389 (M^+^, 5), 263 (100), 247 (99), 195 (30), 167 (27), 77 (25), 41 (50). Anal. Calcd for C_23_H_23_N_3_O_3_: C, 70.93; H, 5.95; N, 10.79; found C, 70.90; H, 5.98; N, 10.68.


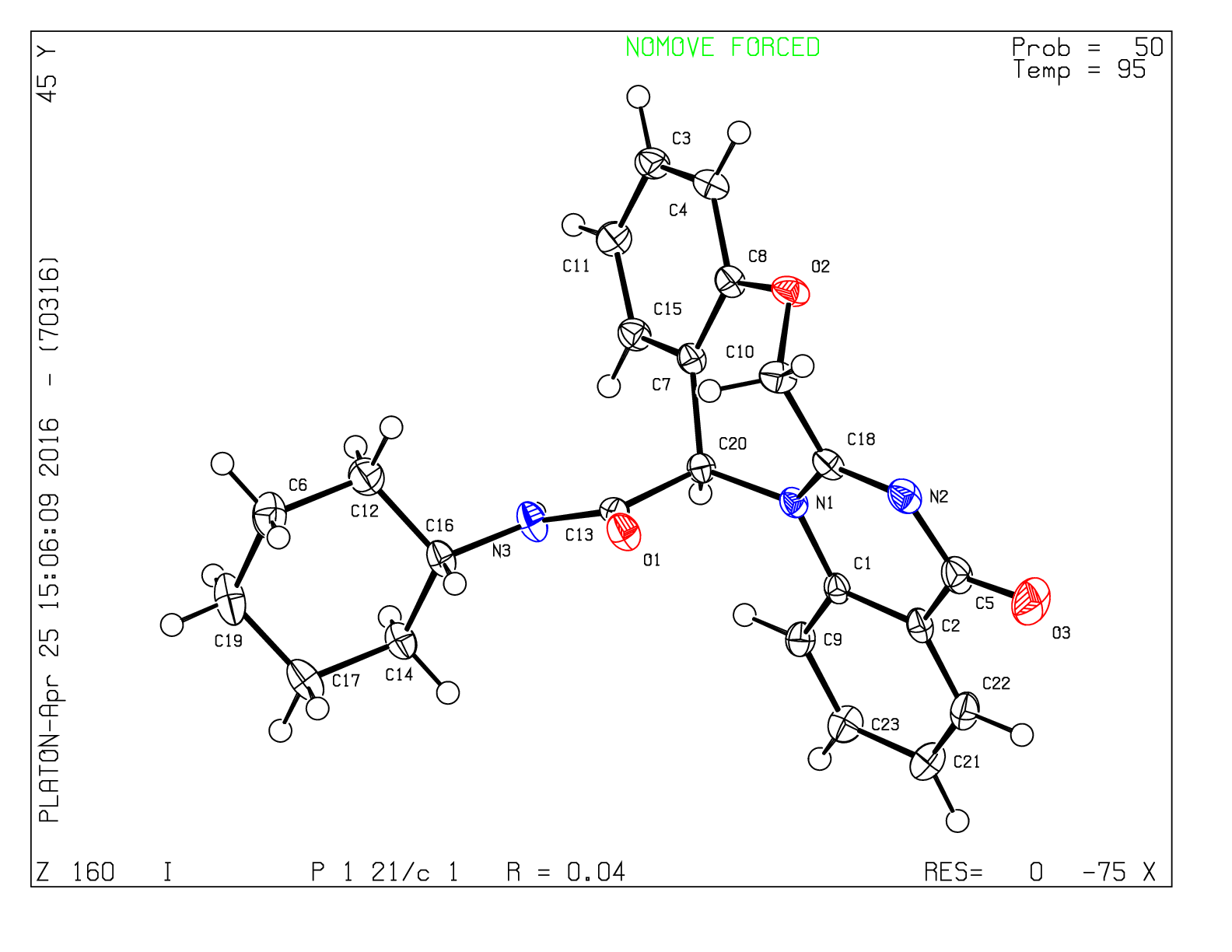


ORTEP diagram for **5c**; Summary of Data: The Cambridge Crystallographic Data Centre (CCDC) 1477764; Unit Cell Parameters: a 16.4035(4) b 7.8172(2) c 16.5650(5) P21/c.

**2-(2-Carbamoylphenyl)-*N*-cyclohexyl-3-oxoisoindoline-1-carboxamide 9.** White powders: 331 mg, 88% yield; mp=220 °C. IR (KBr) cm^-1^: 3400, 3339, 3186, 2934, 2852, 1763, 1731, 1663, 1552. ^1^H NMR (300.13 MHz, DMSO-*d_6_*) δ: 0.85-1.64 (10H, m, 5CH_2_ of cyclohexyl), 3.56 (1H, br s, NH-C*H* of cyclohexyl), 5.92 (1H, s, CH), 7.37-7.93 (8H, m, H-Ar), 8.99 (1H, br s, NH). ^13^C NMR (75.47 MHz, DMSO-*d_6_*) δ: 25.1, 25.5, 32.1, 32.2, 49.3, 61.9, 115.9, 119.9, 120.4, 123.2, 126.7, 128.1, 131.3, 133.3, 134.7, 141.5, 156.3, 160.2, 167.0, 168.1. MS *m/z*: 376 (M^+^-1, 0.1), 249 (100), 130 (25). Anal. Calcd for C_22_H_23_N_3_O_3_: C, 70.01; H, 6.14; N, 11.13; found C, 70.03; H, 6.08; N, 11.04.


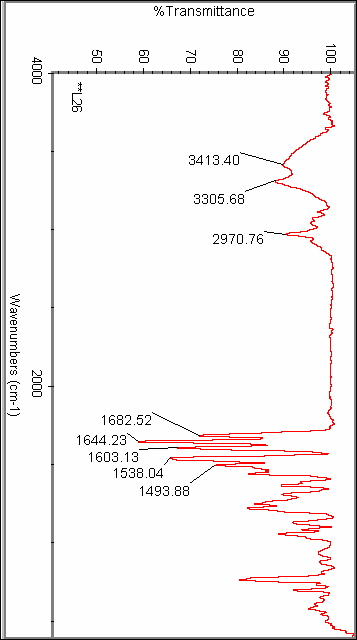


IR of **5a**


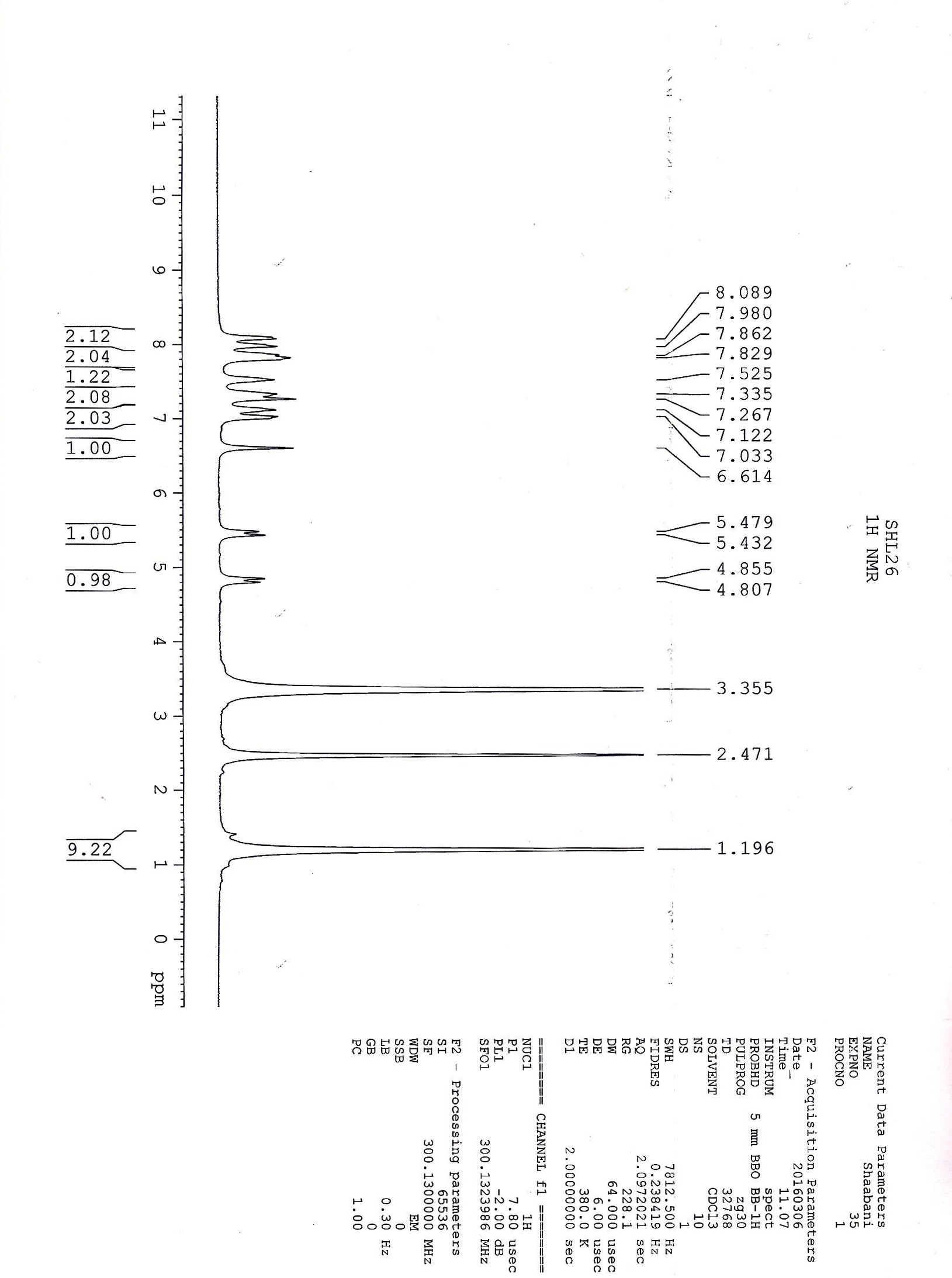


^1^H NMR of **5a**


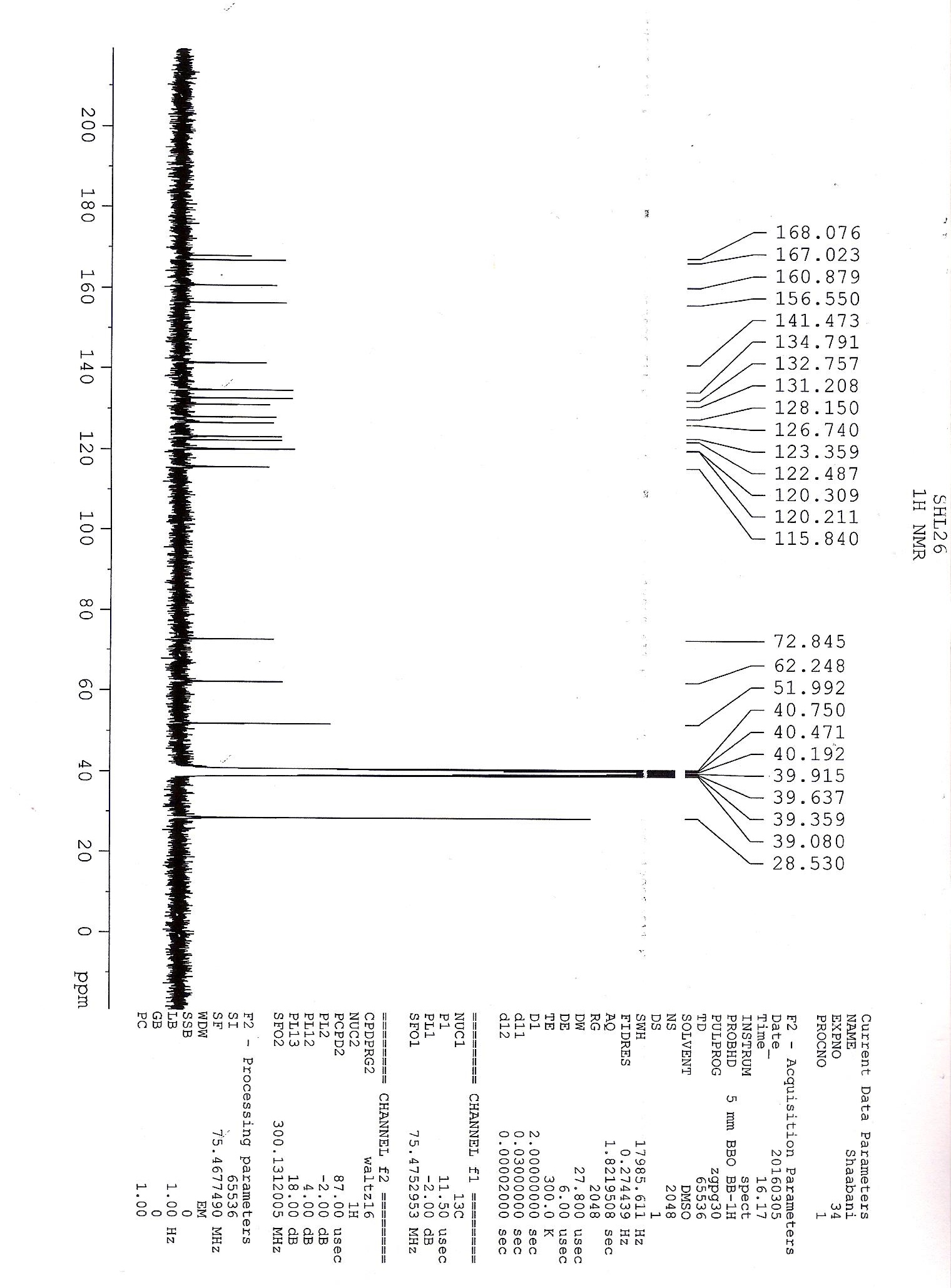


^13^C NMR of **5a**


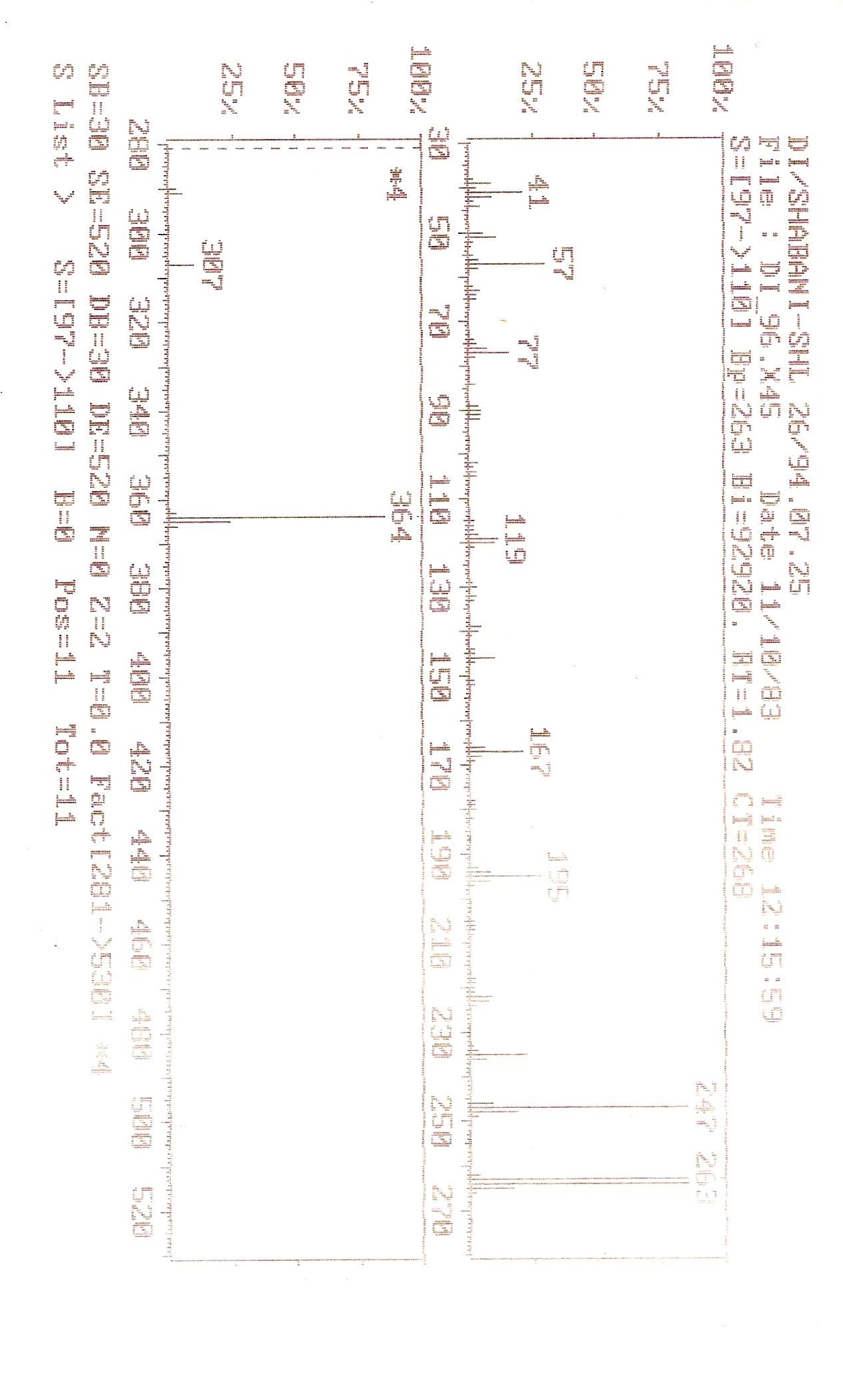


Mass of **5a**


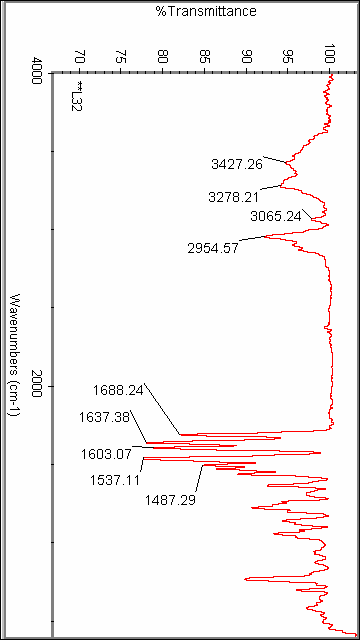


IR of **5b**


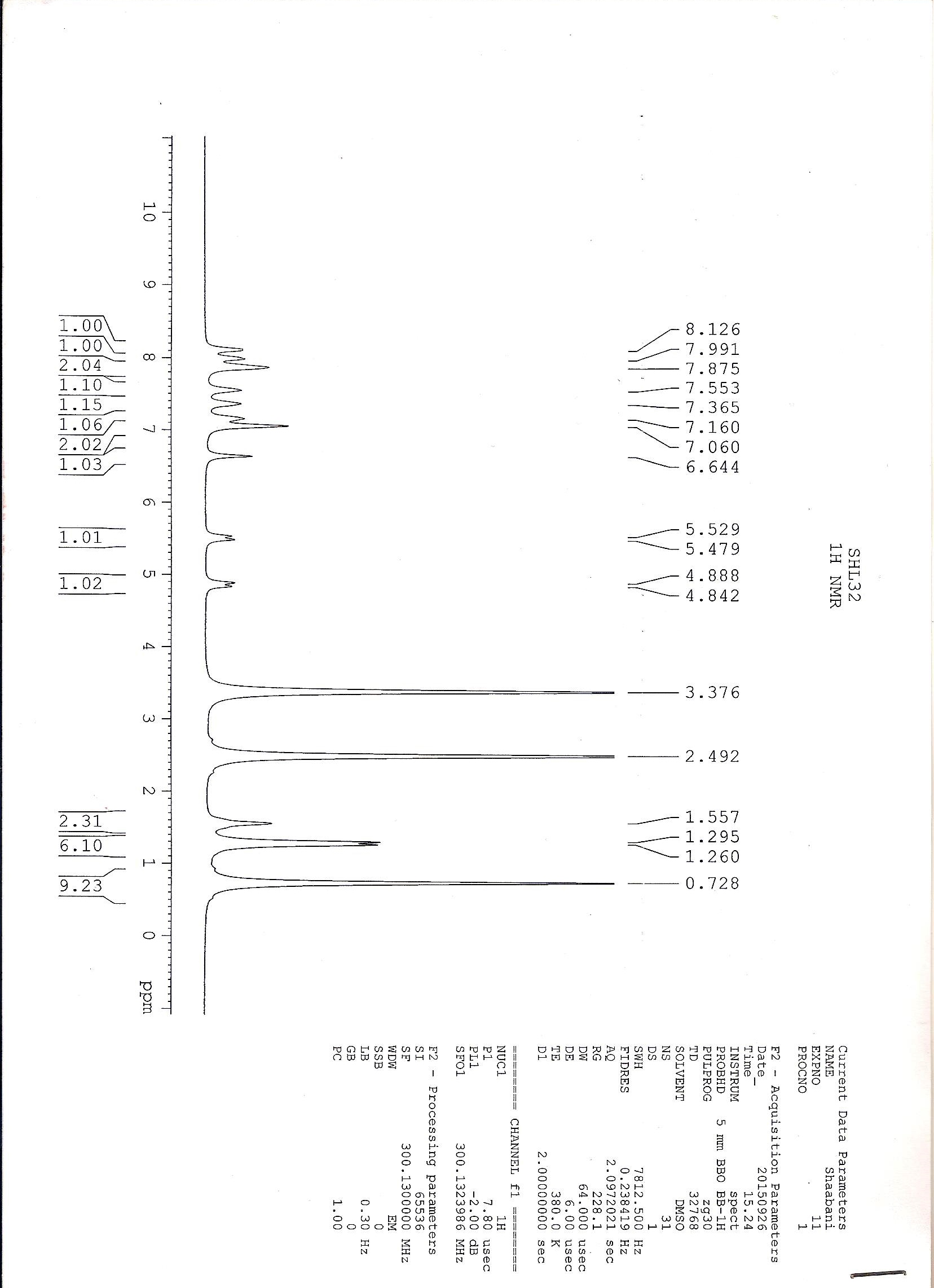


^1^H NMR of **5b**


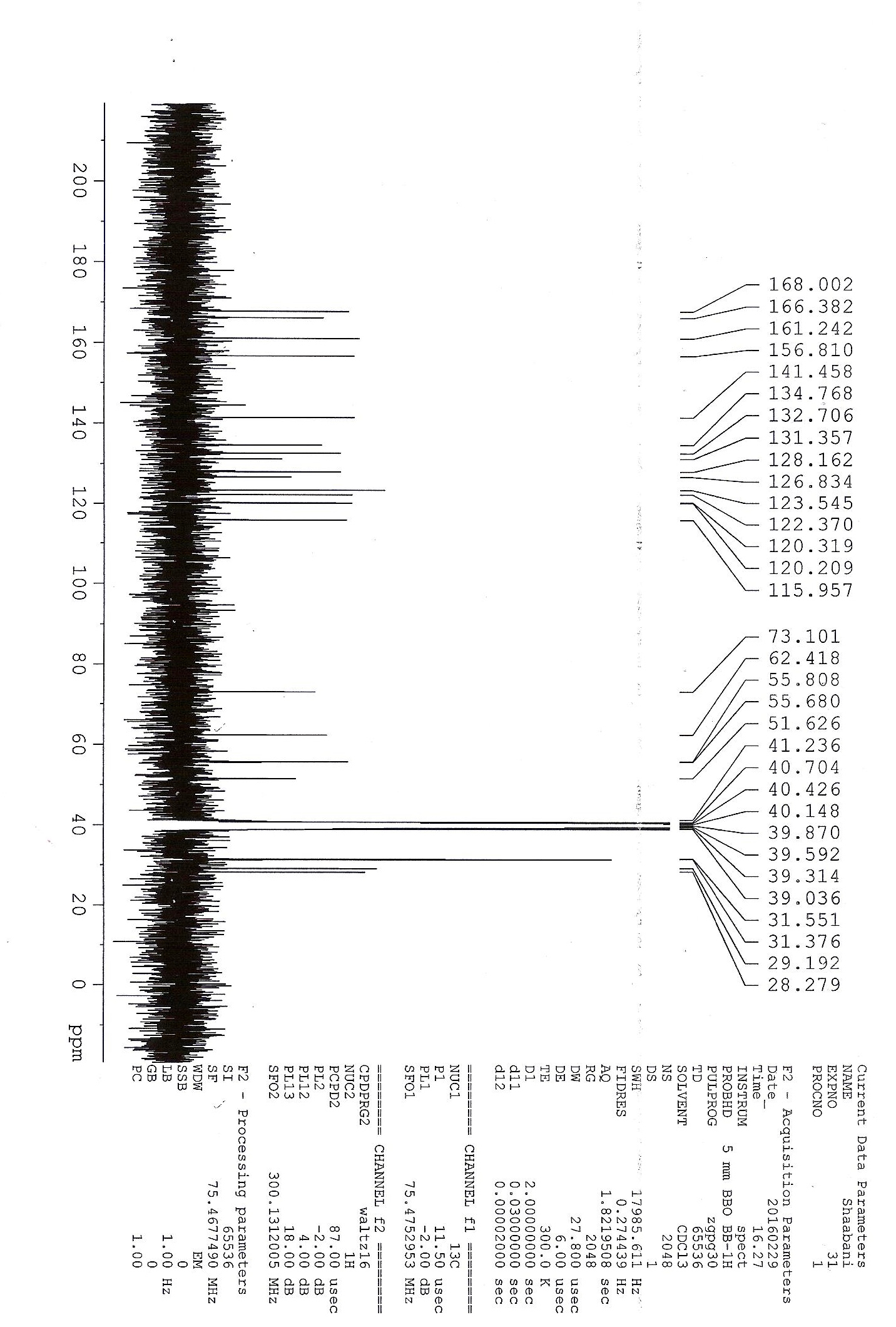


^13^C NMR of **5b**


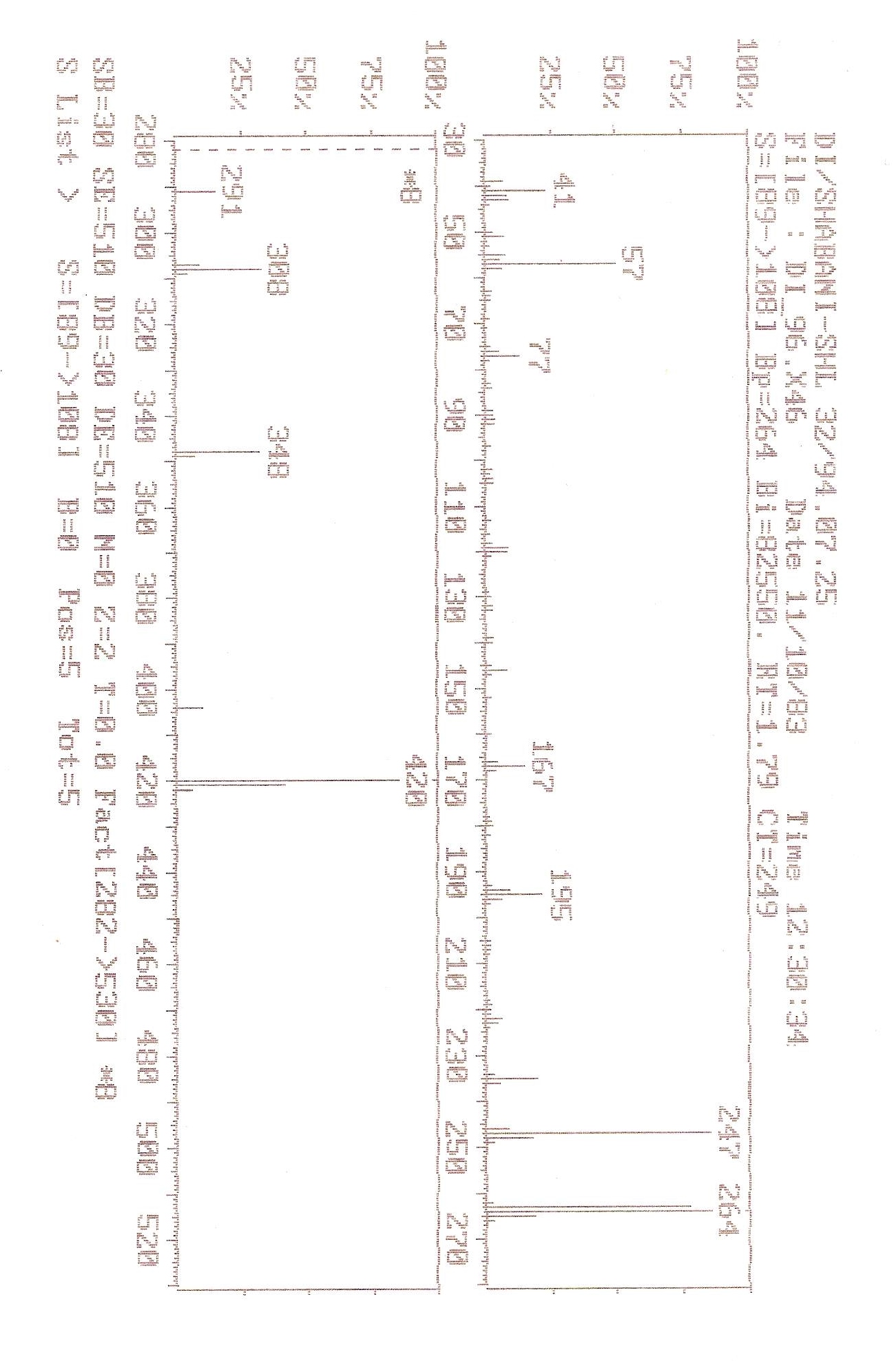


Mass of **5b**


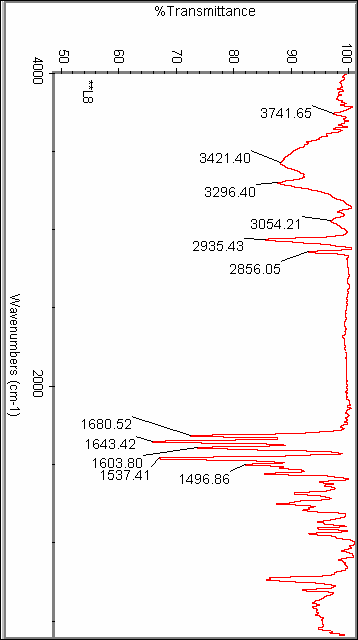


IR of **5c**


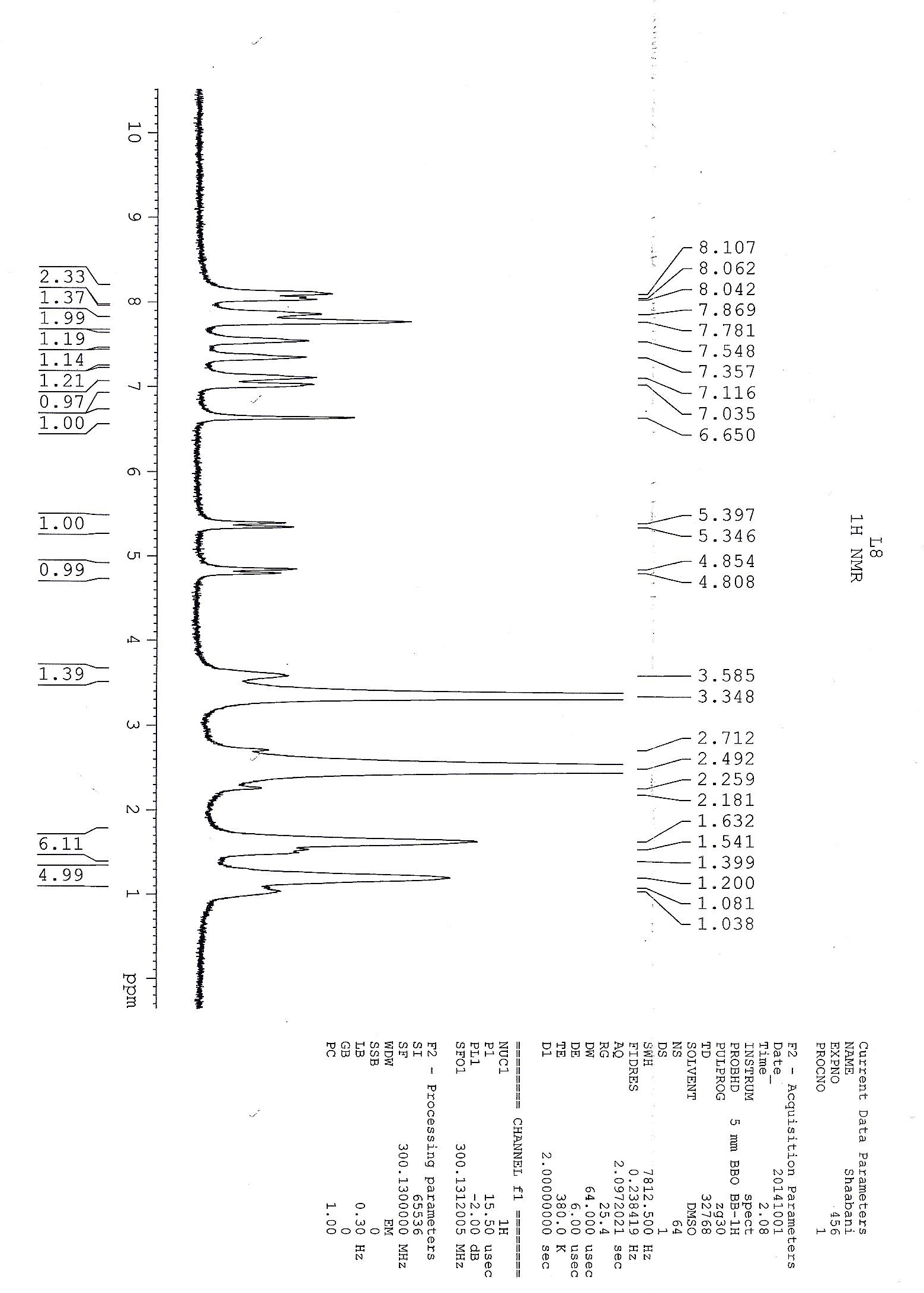


^1^H NMR of **5c**


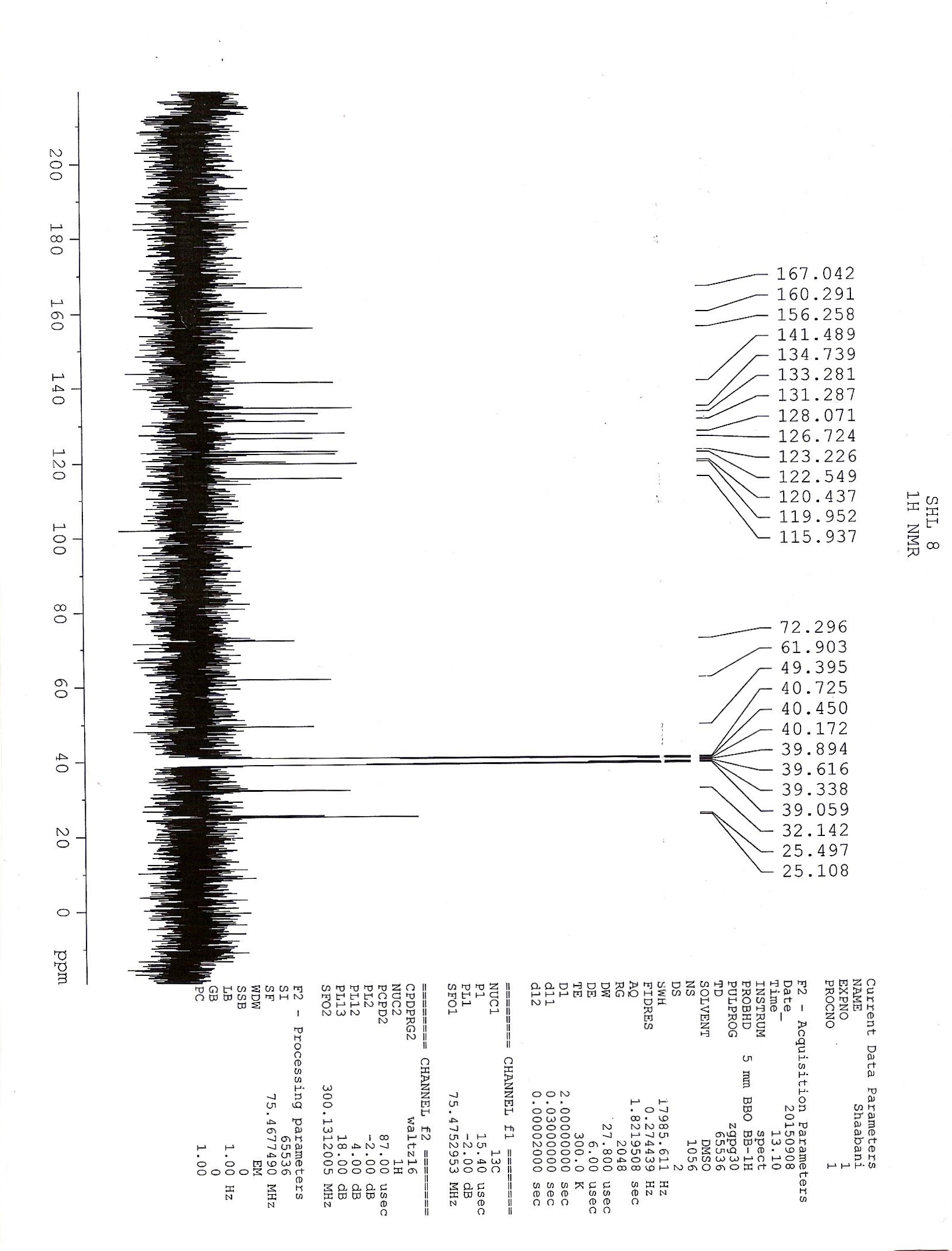


^13^C NMR of **5c**


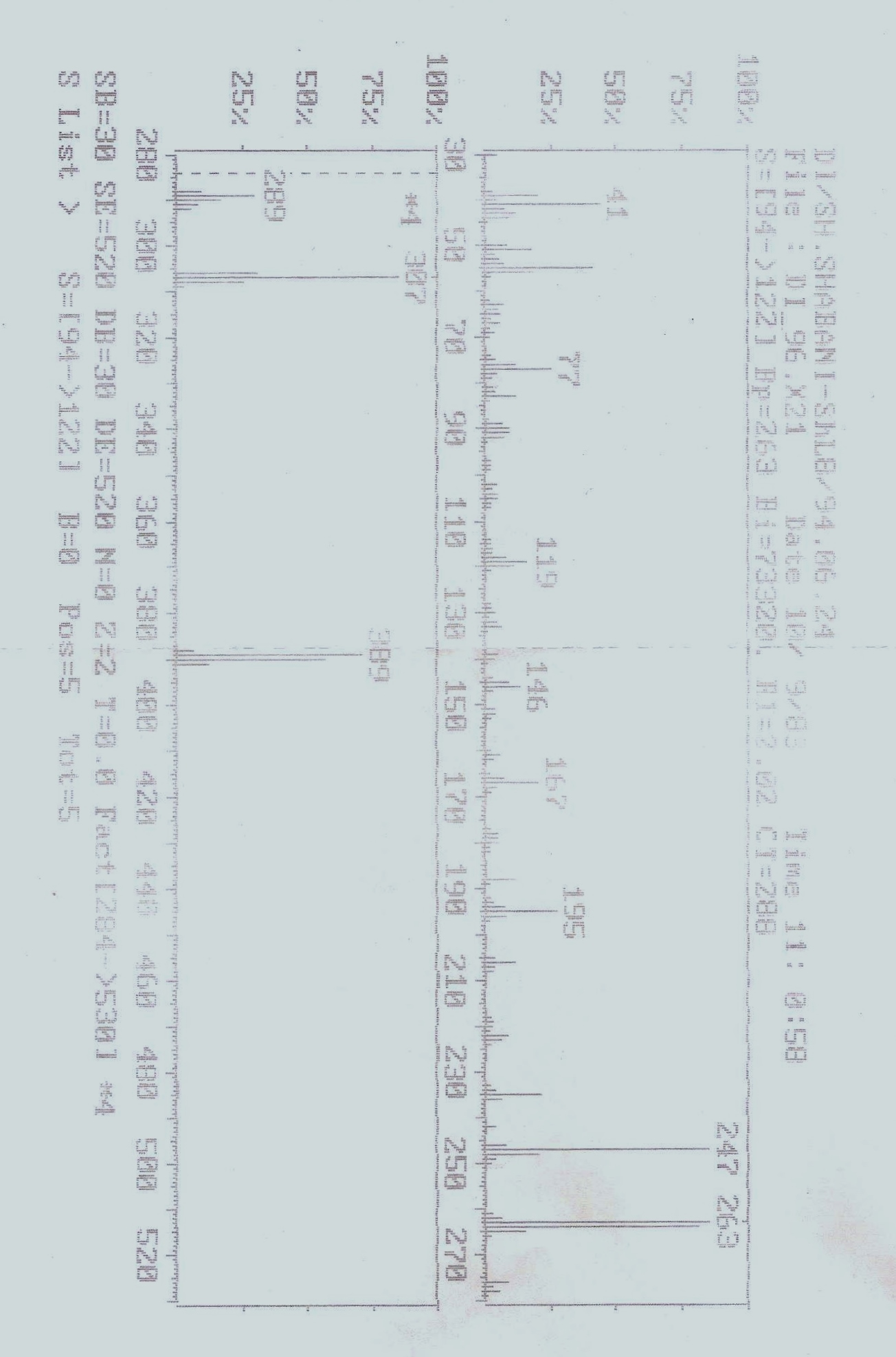


Mass of **5c**

IR of **9**


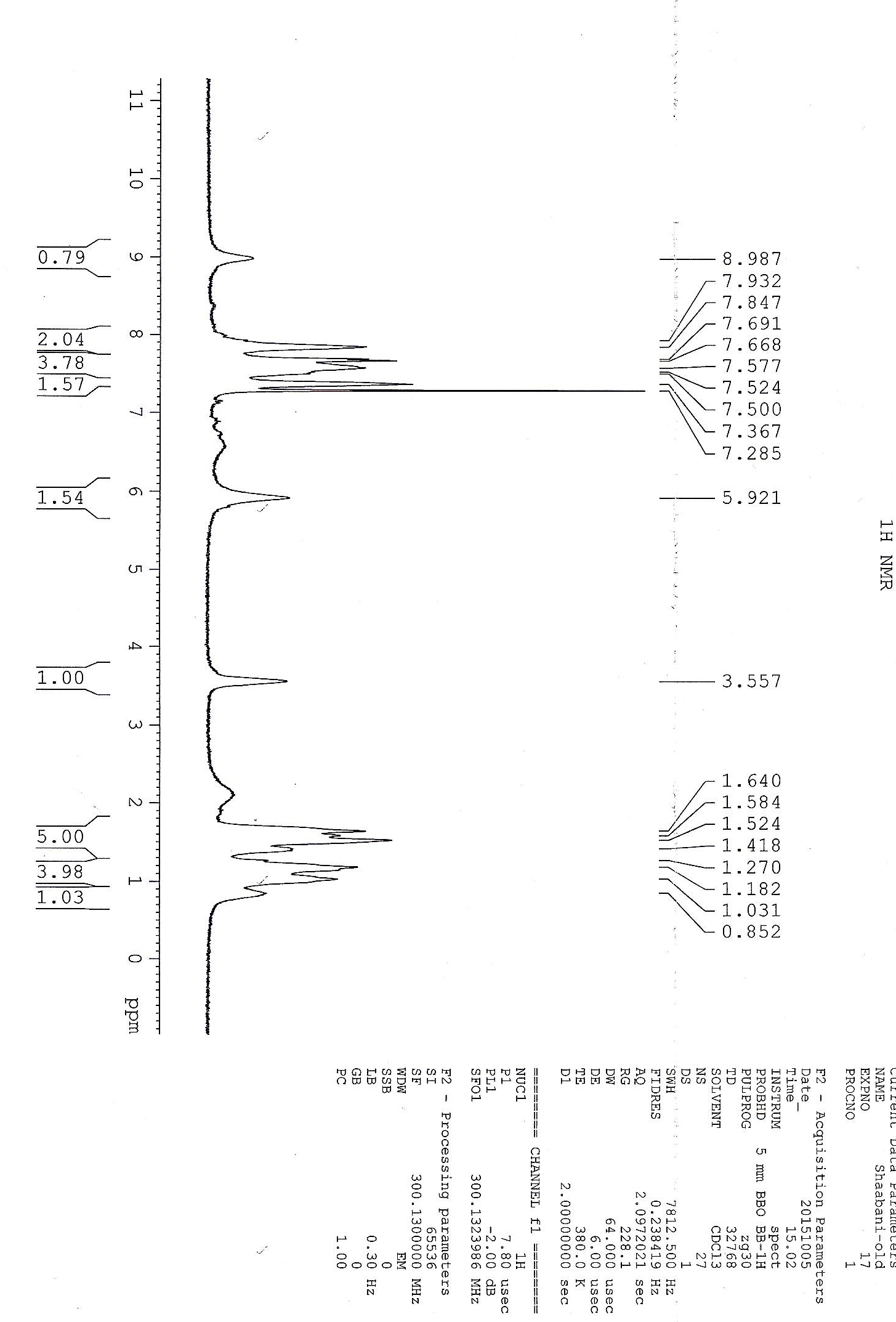


^1^H NMR of **9**


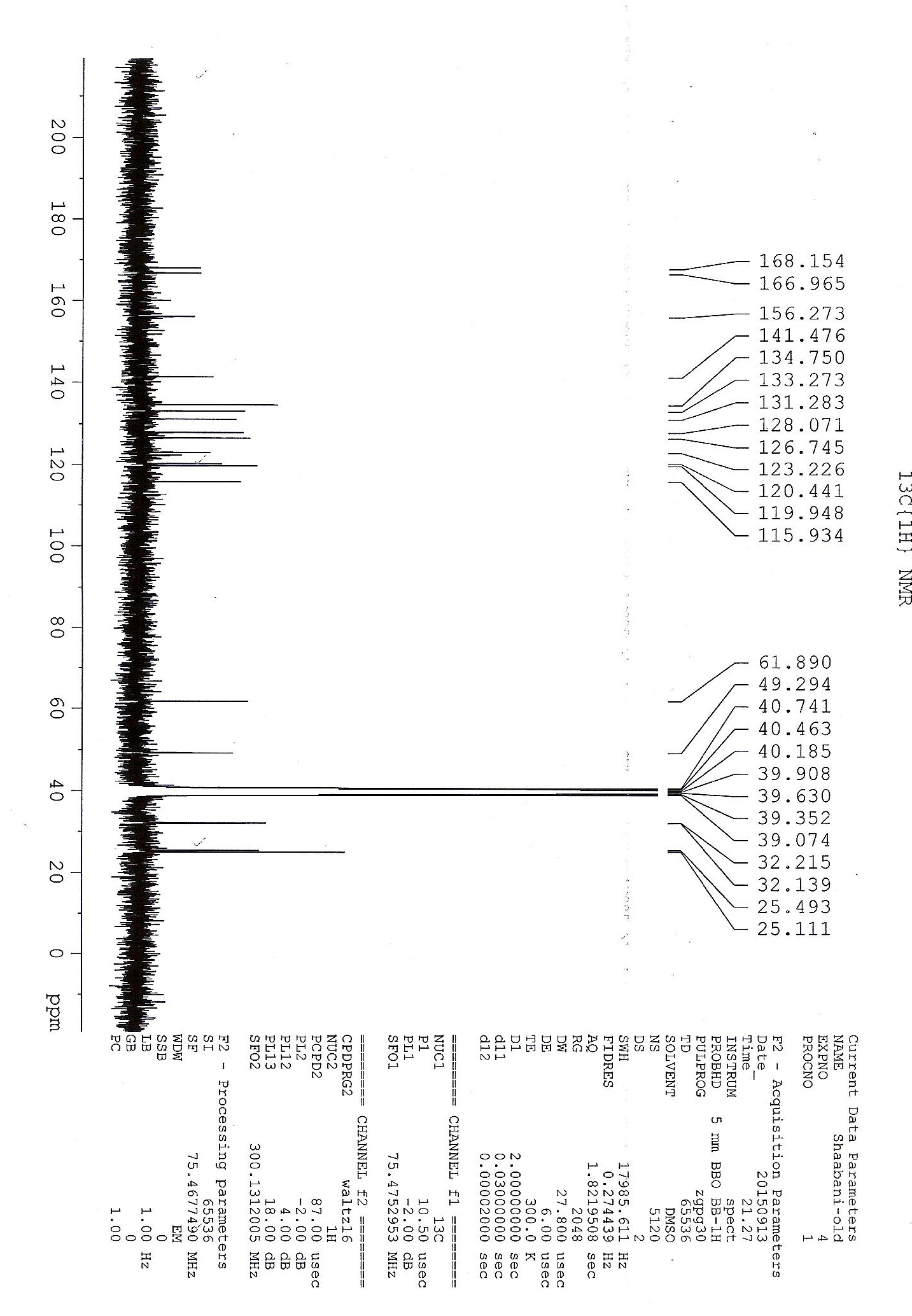


^13^C NMR of **9**


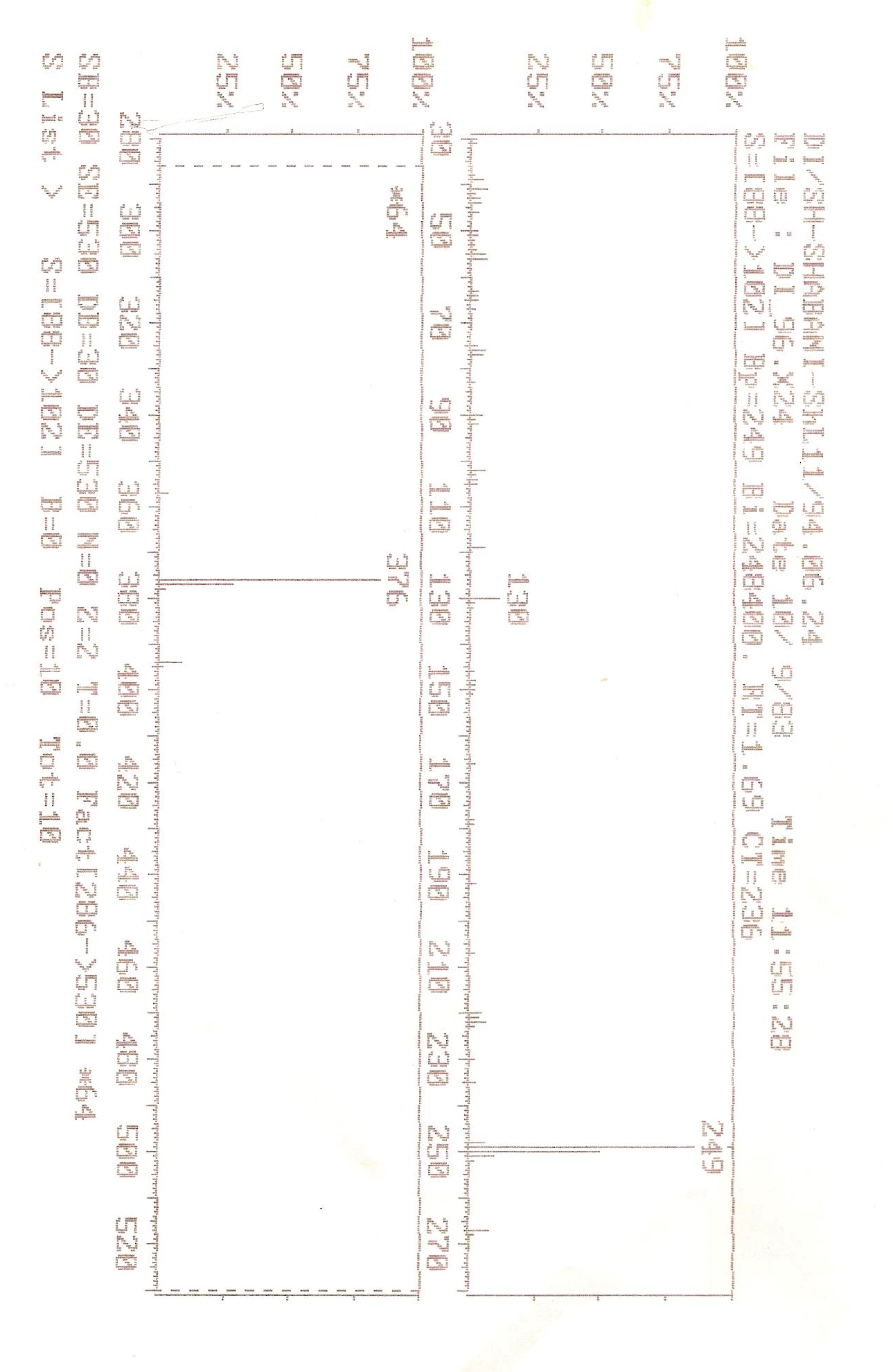


Mass of **9**

**Chemoinformatic analysis**

Sub-structure search for benzoxazepine-quinazolinone, benzoxazepine and quinazolinone was done using two databases, ChEMBL and SciFinder and the results are shown in Table S1. ChEMBL is an open large-scale bioactivity database of small molecules (https://www.ebi.ac.uk/chembl).

**Table S1.** Sbu-structure search results of benzoxazepine-quinazolinone, benzoxazepine and quinazolinone.

| **Database** | **Number of hits** | | |
| --- | --- | --- | --- |
|  | Benzoxazepine-quinazolinone | Benzoxazepine | Quinazolinone |
| **ChEMBL** | 0 | 1206 | 291 |
| **SciFinder** | 0 | 64123 | 119342 |

The graph of molecular weight against AlogP of 1206 benzoxazepines and 291 quinazolinones from ChEMBL are shown in Figure S1. In this figure, the color change is based on RO5 violation.

**
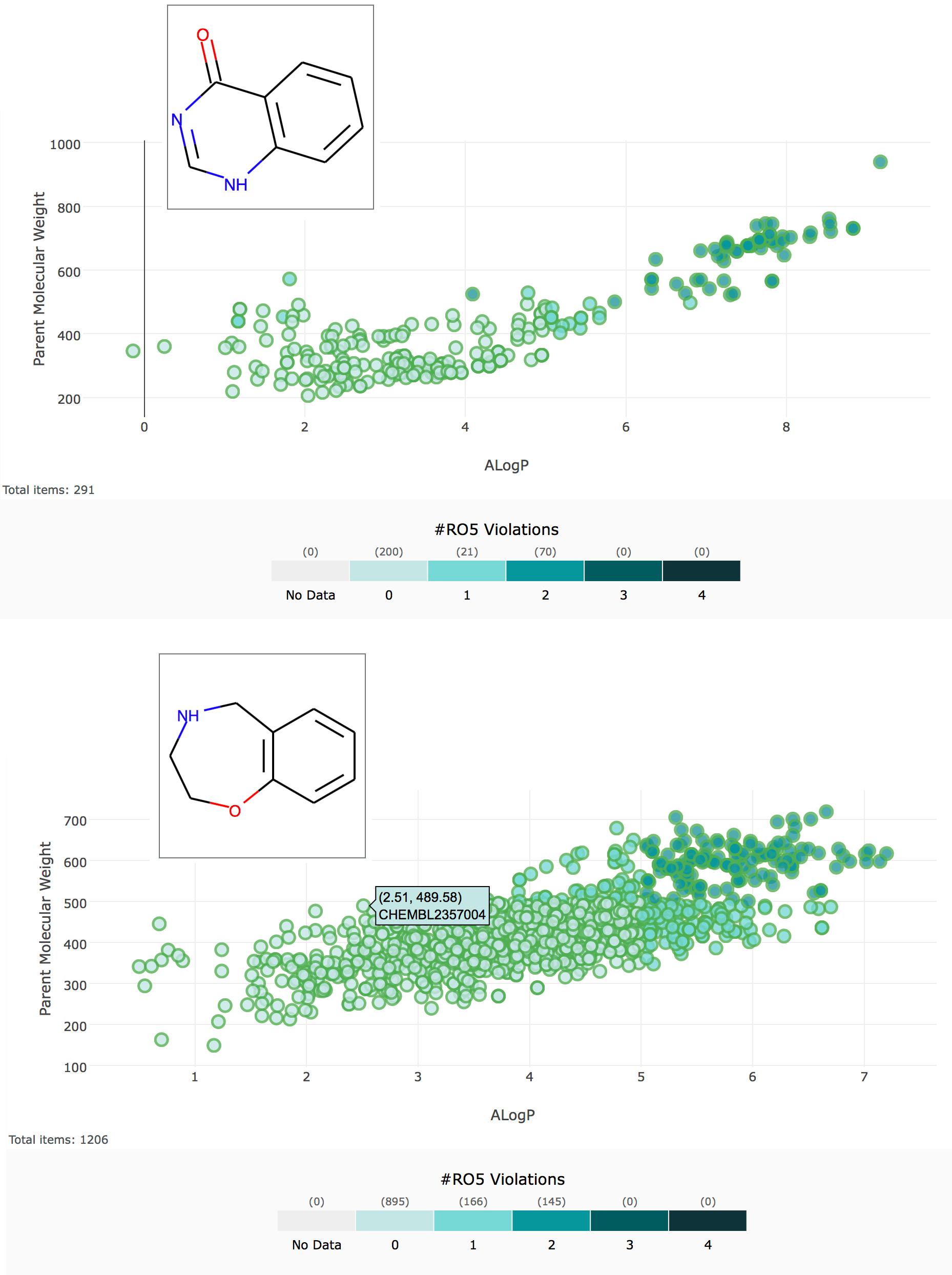
**

**Fig. S1.** MW/ALogP/RO5 violation.

**Virtual library synthesis**

The virtual library of benzoxazepine-quinazolinones was created using 13 2-formylbenzoic acids, 14 2-aminobenzamides and 40 isocyandies as reactants. Therefore, the theoretical chemical space of this virtual library is 13 × 14 × 40 = 7280 (stereoisomers are not included). To investigate such large chemical space, the program RandReactor was used to provide smaller random sublibrary (N=100) as smiles file.^1^ The smiles file was then uploaded into Instant JChem for calculating molecular weight and LogP. This data was exported as an excel file in order to draw MW vs cLogP plot.

1. Huang, Y.; Wolf, S.; Bista, M.; Meireles, L.; Camacho, C.; Holak, T. A.; Dömling, A. Chem. Biol. Drug Des. 2010, 76, 116-129.

**List of 2-formylbenzoic acid smiles:**

C1=CC=C(C(=C1)C=O)OCC(=O)O

CC1=C(C(=CC=C1)C=O)OCC(=O)O

CC1=CC(=C(C=C1)OCC(=O)O)C=O

COC1=CC(=C(C=C1)C=O)OCC(=O)O

CC1=CC(=C(C(=C1)C=O)OCC(=O)O)C

COC1=CC=CC(=C1OCC(=O)O)C=O

COC1=CC(=C(C=C1)OCC(=O)O)C=O

CCOC1=CC=CC(=C1OCC(=O)O)C=O

C1=CC(=C(C=C1Cl)C=O)OCC(=O)O

C1=CC(=C(C=C1Br)C=O)OCC(=O)O

C1=CC(=C(C(=C1)Cl)OCC(=O)O)C=O

COC1=CC(=C(C(=C1)OCC(=O)O)C=O)OC

C1=CC(=C(C=C1[N+](=O)[O-])C=O)OCC(=O)O

**List of 2-aminobenzamides:**

C1=CC=C(C(=C1)C(=O)N)N

CC1=C(C(=CC=C1)N)C(=O)N

CC1=C(C(=CC=C1)C(=O)N)N

CC1=CC(=C(C=C1)C(=O)N)N

C1=CC(=C(C=C1Cl)N)C(=O)N

C1=CC(=C(C=C1Br)N)C(=O)N

C1=CC(=C(C=C1F)N)C(=O)N

CC1=CC(=C(C(=C1)C(=O)N)N)C

C1=CC(=C(C=C1I)N)C(=O)N

C1=CC(=C(C(=C1)Cl)C(=O)N)N

C1=CC(=C(C(=C1)F)C(=O)N)N

C1=CC(=C(C=C1C#N)C(=O)N)N

C1=CC(=C(C=C1Br)C(=O)N)N

COC1=CC(=C(C=C1)C(=O)N)N

**List of isocyanides:**

OCC[N+]#[C-]

[C-]#[N+]CCN1CCOCC1

[C-]#[N+]CCCN1CCOCC1

CN1CCN(CC[N+]#[C-])CC1

CN1CCN(CC[N+]#[C-])CC1

[C-]#[N+]CC1=CC=NC=C1

CC(C)(C)[N+]#[C-]

CCCCN1CCN(CC[N+]#[C-])CC1

CCCCOCCC[N+]#[C-]

COC1=CC([N+]#[C-])=C(OC)C=C1

COC1=CC=CC(=C1)[N+]#[C-]

COC1=CC=C(C=C1)[N+]#[C-]

[C-]#[N+]C1=CC=C(C=C1)C#N

COC1=CC=C(C[N+]#[C-])C=C1

CC(C)CC[N+]#[C-]

CC(C)(C)C[N+]#[C-]

[C-]#[N+]CC1=CC=CC=C1

CCCC(C)[N+]#[C-]

CCCCC[N+]#[C-]

[C-]#[N+]C1CCCCC1

FC1=CC=C(C=C1)[N+]#[C-]

COC1=CC=C(CC[N+]#[C-])C=C1

CCOC1=CC=C(C=C1)[N+]#[C-]

FC1=CC=C(C[N+]#[C-])C=C1

CC(C)(C)CC[N+]#[C-]

CCOC1=CC=C(C[N+]#[C-])C=C1

FC1=CC(F)=C(C=C1)[N+]#[C-]

FC1=CC(F)=C(C[N+]#[C-])C=C1

[C-]#[N+]CCC1=CC=CC=C1

[C-]#[N+]C12CC3CC(CC(C3)C1)C2

FC1=CC=CC=C1CC[N+]#[C-]

CCCCCC[N+]#[C-]

ClC1=CC=CC=C1[N+]#[C-]

ClC1=CC=C(C=C1)[N+]#[C-]

ClC1=CC=C(C[N+]#[C-])C=C1

FC1=C(C=CC(Cl)=C1)[N+]#[C-]

CC(C)CCC(C)[N+]#[C-]

CC1(C)C2CCC1(C)C(C2)[N+]#[C-]

CC(C)(C)CC(C)(C)[N+]#[C-]

CC12CC3CC(C)(C1)CC(C3)(C2)[N+]#[C-]

**List of 100 randomly generated library:**

OCCNC(=O)C1N2C(COC3=C1C=CC=C3)=NC(=O)C1=C2C=CC=C1

CCOC1=CC=C(NC(=O)C2N3C(COC4=C2C=CC=C4)=NC(=O)C2=C3C=C(I)C=C2)C=C1

COC1=CC=CC2=C1OCC1=NC(=O)C3=C(C=CC(=C3)C#N)N1C2C(=O)NC1=CC=C(Cl)C=C1

CC12CC3CC(C)(C1)CC(C3)(C2)NC(=O)C1N2C(COC3=C1C=C(C=C3)[N+]([O-])=O)=NC(=O)C1=C2C=C(Br)C=C1

[O-][N+](=O)C1=CC2=C(OCC3=NC(=O)C4=C(C=CC=C4Cl)N3C2C(=O)NC2CCCCC2)C=C1

COC1=CC(NC(=O)C2N3C(COC4=C2C=C(C=C4)[N+]([O-])=O)=NC(=O)C2=C3C=CC(=C2)C#N)=C(OC)C=C1

CN1CCN(CCNC(=O)C2N3C(COC4=C2C=C(C=C4)[N+]([O-])=O)=NC(=O)C2=C3C=CC(Br)=C2)CC1

CCOC1=CC=C(CNC(=O)C2N3C(COC4=C2C=C(OC)C=C4)=NC(=O)C2=C3C=CC=C2)C=C1

FC1=C(NC(=O)C2N3C(COC4=C2C=C(Cl)C=C4)=NC(=O)C2=C3C=CC=C2)C=CC(Cl)=C1

COC1=CC2=C(C=C1)C(N1C(CO2)=NC(=O)C2=C1C=CC=C2)C(=O)NC1=CC=C(F)C=C1

COC1=CC=CC(NC(=O)C2N3C(COC4=C2C=CC=C4C)=NC(=O)C2=C3C=CC=C2)=C1

O=C(NCC1=CC=NC=C1)C1N2C(COC3=C1C=CC=C3)=NC(=O)C1=C2C=CC=C1

CCOC1=CC=C(CNC(=O)C2N3C(COC4=C2C=CC(OC)=C4)=NC(=O)C2=C3C=CC=C2)C=C1

CC1=CC2=C(N3C(C(=O)NC4=CC=C(C=C4)C#N)C4=C(OCC3=NC2=O)C(C)=CC=C4)C(C)=C1

CCOC1=CC=CC2=C1OCC1=NC(=O)C3=C(C=CC(=C3)C#N)N1C2C(=O)NCCC1=CC=CC=C1

CC(C)CCC(C)NC(=O)C1N2C(COC3=C1C=CC=C3Cl)=NC(=O)C1=C2C=CC(=C1)C#N

CCCCCNC(=O)C1N2C(COC3=C1C(OC)=CC(OC)=C3)=NC(=O)C1=C2C=C(Br)C=C1

CCCCCCNC(=O)C1N2C(COC3=C1C=CC=C3C)=NC(=O)C1=C2C=CC=C1Cl

CCOC1=CC=CC2=C1OCC1=NC(=O)C3=C(C=C(Br)C=C3)N1C2C(=O)NC(C)(C)CC(C)(C)C

COC1=CC=C(CCNC(=O)C2N3C(COC4=C2C=C(Cl)C=C4)=NC(=O)C2=C3C=CC=C2Cl)C=C1

COC1=CC=CC(NC(=O)C2N3C(COC4=C2C=CC=C4Cl)=NC(=O)C2=C3C=CC(=C2)C#N)=C1

COC1=CC2=C(C(N3C(CO2)=NC(=O)C2=C3C=C(Br)C=C2)C(=O)NCC2=CC=NC=C2)C(OC)=C1

CCOC1=CC=C(CNC(=O)C2N3C(COC4=C2C=C(C=C4)[N+]([O-])=O)=NC(=O)C2=C3C=CC=C2C)C=C1

CC1=CC2=C(N3C(C(=O)NC4=CC=C(C=C4)C#N)C4=C(OCC3=NC2=O)C=CC(=C4)[N+]([O-])=O)C(C)=C1

CC1=CC2=C(C=C1)C(=O)N=C1COC3=C(C=C(C=C3)[N+]([O-])=O)C(N21)C(=O)NC(C)(C)C

CN1CCN(CCNC(=O)C2N3C(COC4=C2C=C(C=C4)[N+]([O-])=O)=NC(=O)C2=C3C=CC=C2C)CC1

CC1=CC2=C(N3C(C(=O)NCCN4CCOCC4)C4=C(OCC3=NC2=O)C=CC(=C4)[N+]([O-])=O)C(C)=C1

OCCNC(=O)C1N2C(COC3=C1C=C(C=C3)[N+]([O-])=O)=NC(=O)C1=C2C=CC=C1F

CCOC1=CC=C(NC(=O)C2N3C(COC4=C2C=C(Br)C=C4)=NC(=O)C2=C3C=CC(Br)=C2)C=C1

COC1=CC=C(NC(=O)C2N3C(COC4=C2C=C(C)C=C4C)=NC(=O)C2=C3C=C(F)C=C2)C=C1

CC1=CC2=C(OCC3=NC(=O)C4=C(C=C(Cl)C=C4)N3C2C(=O)NCCC2=CC=CC=C2)C=C1

CC(C)CCC(C)NC(=O)C1N2C(COC3=C1C=CC=C3C)=NC(=O)C1=C2C=C(I)C=C1

CCCCCNC(=O)C1N2C(COC3=C1C=CC=C3)=NC(=O)C1=C2C=C(Cl)C=C1

CCCCCCNC(=O)C1N2C(COC3=C1C=CC=C3)=NC(=O)C1=C2C=CC=C1F

COC1=CC=CC2=C1OCC1=NC(=O)C3=C(C=C(Br)C=C3)N1C2C(=O)NC(C)(C)CC(C)(C)C

[O-][N+](=O)C1=CC2=C(OCC3=NC(=O)C4=C(C=CC(=C4)C#N)N3C2C(=O)NC2=CC=C(F)C=C2)C=C1

COC1=CC=CC(NC(=O)C2N3C(COC4=C2C=C(C=C4)[N+]([O-])=O)=NC(=O)C2=C3C=C(Br)C=C2)=C1

CC1=CC=CC2=C1N1C(C(=O)NCC3=CC=NC=C3)C3=C(OCC1=NC2=O)C=CC(=C3)[N+]([O-])=O

CCOC1=CC=C(CNC(=O)C2N3C(COC4=C2C=CC(OC)=C4)=NC(=O)C2=C3C=CC=C2C)C=C1

COC1=CC2=C(C=C1)C(N1C(CO2)=NC(=O)C2=C1C=CC=C2)C(=O)NC1=C(F)C=C(Cl)C=C1

CCCC(C)NC(=O)C1N2C(COC3=C1C=CC=C3C)=NC(=O)C1=C2C(C)=CC(C)=C1

CCCCCCNC(=O)C1N2C(COC3=C1C=C(C)C=C3)=NC(=O)C1=C2C=C(C)C=C1

CCOC1=CC=CC2=C1OCC1=NC(=O)C3=C(C=C(I)C=C3)N1C2C(=O)NCC(C)(C)C

CCCCN1CCN(CCNC(=O)C2N3C(COC4=C2C=CC(OC)=C4)=NC(=O)C2=C3C=CC(=C2)C#N)CC1

FC1=CC(F)=C(NC(=O)C2N3C(COC4=C2C=CC=C4)=NC(=O)C2=C3C=C(F)C=C2)C=C1

COC1=CC2=C(C=C1)C(N1C(CO2)=NC(=O)C2=C1C=CC=C2F)C(=O)NC1=C(F)C=C(Cl)C=C1

CCCC(C)NC(=O)C1N2C(COC3=C1C=CC=C3C)=NC(=O)C1=C2C=CC(Br)=C1

CCCCOCCCNC(=O)C1N2C(COC3=C1C=CC=C3)=NC(=O)C1=C2C=C(OC)C=C1

COC1=CC2=C(OCC3=NC(=O)C4=C(C=C(F)C=C4)N3C2C(=O)NCCN2CCN(C)CC2)C=C1

CC(C)(C)CCNC(=O)C1N2C(COC3=C1C=C(Cl)C=C3)=NC(=O)C1=C2C=CC=C1F

CC1=CC2=C(OCC3=NC(=O)C4=C(C=CC(=C4)C#N)N3C2C(=O)NCC2=CC=C(Cl)C=C2)C(C)=C1

CCCC(C)NC(=O)C1N2C(COC3=C1C=C(Cl)C=C3)=NC(=O)C1=C2C=C(Br)C=C1

CCCCOCCCNC(=O)C1N2C(COC3=C1C=CC=C3Cl)=NC(=O)C1=C2C=CC=C1Cl

COC1=CC2=C(C(N3C(CO2)=NC(=O)C2=C3C=C(Cl)C=C2)C(=O)NCCN2CCN(C)CC2)C(OC)=C1

COC1=CC=CC2=C1OCC1=NC(=O)C3=C(N1C2C(=O)NCCCN1CCOCC1)C(C)=CC=C3

CC1=CC2=C(OCC3=NC(=O)C4=C(C=CC=C4C)N3C2C(=O)NCCN2CCOCC2)C=C1

CCOC1=CC=CC2=C1OCC1=NC(=O)C3=C(N1C2C(=O)NCCO)C(C)=CC(C)=C3

CCOC1=CC=C(NC(=O)C2N3C(COC4=C2C=C(Cl)C=C4)=NC(=O)C2=C3C=CC(=C2)C#N)C=C1

COC1=CC=C(NC(=O)C2N3C(COC4=C2C=CC=C4Cl)=NC(=O)C2=C3C=C(Br)C=C2)C=C1

COC1=CC2=C(C(N3C(CO2)=NC(=O)C2=C3C=CC=C2Cl)C(=O)NCC2=CC=NC=C2)C(OC)=C1

CCOC1=CC=C(CNC(=O)C2N3C(COC4=C2C=C(C)C=C4)=NC(=O)C2=C3C=C(Br)C=C2)C=C1

CCOC1=CC=CC2=C1OCC1=NC(=O)C3=C(C=CC=C3Cl)N1C2C(=O)NC1=CC=C(C=C1)C#N

ClC1=CC=CC2=C1OCC1=NC(=O)C3=C(C=CC(Br)=C3)N1C2C(=O)NCCC1=CC=CC=C1

COC1=CC2=C(C=C1)C(=O)N=C1COC3=C(C=C(Cl)C=C3)C(N21)C(=O)NC(C)CCC(C)C

CCCCCNC(=O)C1N2C(COC3=C1C=CC=C3Cl)=NC(=O)C1=C2C=C(F)C=C1

CCCCCCNC(=O)C1N2C(COC3=C1C=CC=C3OC)=NC(=O)C1=C2C=CC(=C1)C#N

CC(C)(C)CC(C)(C)NC(=O)C1N2C(COC3=C1C=C(Br)C=C3)=NC(=O)C1=C2C=C(Cl)C=C1

CC1=CC2=C(OCC3=NC(=O)C4=C(N3C2C(=O)NC2CCCCC2)C(C)=CC=C4)C(C)=C1

CC1=CC2=C(OCC3=NC(=O)C4=C(N3C2C(=O)NC2=CC=CC=C2Cl)C(C)=CC=C4)C=C1

COC1=CC2=C(C=C1)C(N1C(CO2)=NC(=O)C2=C1C(C)=CC(C)=C2)C(=O)NC(C)(C)CC(C)(C)C

FC1=CC=C(NC(=O)C2N3C(COC4=C2C=C(Br)C=C4)=NC(=O)C2=C3C=CC(Br)=C2)C=C1

COC1=CC=CC(NC(=O)C2N3C(COC4=C2C=C(C)C=C4C)=NC(=O)C2=C3C=C(F)C=C2)=C1

COC1=CC=CC2=C1OCC1=NC(=O)C3=C(C=CC=C3F)N1C2C(=O)NCC1=C(F)C=C(F)C=C1

COC1=CC=C(CNC(=O)C2N3C(COC4=C2C=C(C)C=C4)=NC(=O)C2=C3C=CC(Br)=C2)C=C1

COC1=CC2=C(C=C1)C(N1C(CO2)=NC(=O)C2=C1C=C(Br)C=C2)C(=O)NC12CC3CC(CC(C3)C1)C2

COC1=CC2=C(OCC3=NC(=O)C4=C(C=C(C)C=C4)N3C2C(=O)NC2CC3CCC2(C)C3(C)C)C=C1

CCCCCNC(=O)C1N2C(COC3=C1C=C(Br)C=C3)=NC(=O)C1=C2C=C(I)C=C1

COC1=CC(NC(=O)C2N3C(COC4=C2C=C(C)C=C4C)=NC(=O)C2=C3C=C(Cl)C=C2)=C(OC)C=C1

CC1=CC2=C(OCC3=NC(=O)C4=C(C=CC=C4C)N3C2C(=O)NCC2=C(F)C=C(F)C=C2)C=C1

CCOC1=CC=CC2=C1OCC1=NC(=O)C3=C(N1C2C(=O)NCC1=CC=C(OC)C=C1)C(C)=CC(C)=C3

ClC1=CC2=C(C=C1)C(=O)N=C1COC3=C(C=CC=C3Cl)C(N21)C(=O)NC12CC3CC(CC(C3)C1)C2

CCOC1=CC=CC2=C1OCC1=NC(=O)C3=C(C=CC=C3C)N1C2C(=O)NC1CC2CCC1(C)C2(C)C

CC1=CC2=C(N3C(C(=O)NC4=CC=C(F)C=C4)C4=C(OCC3=NC2=O)C(Cl)=CC=C4)C(C)=C1

COC1=CC=CC(NC(=O)C2N3C(COC4=C2C(OC)=CC(OC)=C4)=NC(=O)C2=C3C=C(C)C=C2)=C1

CC1=CC2=C(OCC3=NC(=O)C4=C(C=CC=C4Cl)N3C2C(=O)NCC2=C(F)C=C(F)C=C2)C=C1

CCOC1=CC=CC2=C1OCC1=NC(=O)C3=C(C=CC(=C3)C#N)N1C2C(=O)NCC1=CC=C(OC)C=C1

CCOC1=CC=CC2=C1OCC1=NC(=O)C3=C(C=C(Br)C=C3)N1C2C(=O)NC12CC3CC(CC(C3)C1)C2

CC1=CC2=C(C=C1)C(=O)N=C1COC3=C(C=C(Cl)C=C3)C(N21)C(=O)NC1CC2CCC1(C)C2(C)C

CCCCCNC(=O)C1N2C(COC3=C1C=CC=C3Cl)=NC(=O)C1=C2C=C(I)C=C1

CCCCCCNC(=O)C1N2C(COC3=C1C=CC=C3OC)=NC(=O)C1=C2C=CC=C1F

CC1=CC2=C(OCC3=NC(=O)C4=C(C=CC(Br)=C4)N3C2C(=O)NCC(C)(C)C)C=C1

COC1=CC2=C(OCC3=NC(=O)C4=C(C=C(F)C=C4)N3C2C(=O)NCCC2=CC=CC=C2F)C=C1

CC1(C)C2CCC1(C)C(C2)NC(=O)C1N2C(COC3=C1C=C(Cl)C=C3)=NC(=O)C1=C2C=CC=C1F

CCOC1=CC=C(NC(=O)C2N3C(COC4=C2C=C(OC)C=C4)=NC(=O)C2=C3C=C(C)C=C2)C=C1

COC1=CC=C(NC(=O)C2N3C(COC4=C2C=C(Br)C=C4)=NC(=O)C2=C3C=CC=C2C)C=C1

COC1=CC2=C(C=C1)C(N1C(CO2)=NC(=O)C2=C1C=CC=C2C)C(=O)NCCC1=CC=CC=C1

CC(C)CCC(C)NC(=O)C1N2C(COC3=C1C=CC=C3Cl)=NC(=O)C1=C2C=C(F)C=C1

COC1=CC=C(CCNC(=O)C2N3C(COC4=C2C=CC(OC)=C4)=NC(=O)C2=C3C=C(OC)C=C2)C=C1

ClC1=CC=C(NC(=O)C2N3C(COC4=C2C=C(Cl)C=C4)=NC(=O)C2=C3C=C(Br)C=C2)C=C1

ClC1=CC=CC2=C1OCC1=NC(=O)C3=C(C=CC=C3Cl)N1C2C(=O)NCC1=CC=CC=C1
